# Evolutionary trajectory of diverse SARS-CoV-2 variants at the beginning of COVID-19 outbreak

**DOI:** 10.1093/ve/veae020

**Published:** 2024-03-05

**Authors:** Jia-Xin Lv, Xiang Liu, Yuan-Yuan Pei, Zhi-Gang Song, Xiao Chen, Shu-Jian Hu, Jia-Lei She, Yi Liu, Yan-Mei Chen, Yong-Zhen Zhang

**Affiliations:** State Key Laboratory of Genetic Engineering, Greater Bay Area Institute of Precision Medicine (Guangzhou), School of Life Sciences and Human Phenome Institute, Fudan University, No. 2005 Songhu Road, Yangpu District, Shanghai 200438, China; State Key Laboratory of Genetic Engineering, Greater Bay Area Institute of Precision Medicine (Guangzhou), School of Life Sciences and Human Phenome Institute, Fudan University, No. 2005 Songhu Road, Yangpu District, Shanghai 200438, China; State Key Laboratory of Genetic Engineering, Greater Bay Area Institute of Precision Medicine (Guangzhou), School of Life Sciences and Human Phenome Institute, Fudan University, No. 2005 Songhu Road, Yangpu District, Shanghai 200438, China; Shanghai Public Health Clinical Center, No. 2901 Canglang Road, Jinshan District, Shanghai 210508, China; State Key Laboratory of Genetic Engineering, Greater Bay Area Institute of Precision Medicine (Guangzhou), School of Life Sciences and Human Phenome Institute, Fudan University, No. 2005 Songhu Road, Yangpu District, Shanghai 200438, China; Shanghai Public Health Clinical Center, No. 2901 Canglang Road, Jinshan District, Shanghai 210508, China; College of Marine Sciences, South China Agricultural University, No. 483 Wushan Road, Tianhe District, Guangzhou, Guangdong 510642, China; State Key Laboratory of Genetic Engineering, Greater Bay Area Institute of Precision Medicine (Guangzhou), School of Life Sciences and Human Phenome Institute, Fudan University, No. 2005 Songhu Road, Yangpu District, Shanghai 200438, China; Shanghai Public Health Clinical Center, No. 2901 Canglang Road, Jinshan District, Shanghai 210508, China; Shanghai Public Health Clinical Center, No. 2901 Canglang Road, Jinshan District, Shanghai 210508, China; State Key Laboratory of Genetic Engineering, Greater Bay Area Institute of Precision Medicine (Guangzhou), School of Life Sciences and Human Phenome Institute, Fudan University, No. 2005 Songhu Road, Yangpu District, Shanghai 200438, China; State Key Laboratory of Genetic Engineering, Greater Bay Area Institute of Precision Medicine (Guangzhou), School of Life Sciences and Human Phenome Institute, Fudan University, No. 2005 Songhu Road, Yangpu District, Shanghai 200438, China

**Keywords:** COVID-19, SARS-CoV-2, Outbreak, Evolutionary trajectory, Mutations

## Abstract

Despite extensive scientific efforts directed toward the evolutionary trajectory of severe acute respiratory syndrome coronavirus 2 (SARS-CoV-2) in humans at the beginning of the COVID-19 epidemic, it remains unclear how the virus jumped into and evolved in humans so far. Herein, we recruited almost all adult coronavirus disease 2019 (COVID-19) cases appeared locally or imported from abroad during the first 8 months of the outbreak in Shanghai. From these patients, SARS-CoV-2 genomes occupying the important phylogenetic positions in the virus phylogeny were recovered. Phylogenetic and mutational landscape analyses of viral genomes recovered here and those collected in and outside of China revealed that all known SARS-CoV-2 variants exhibited the evolutionary continuity despite the co-circulation of multiple lineages during the early period of the epidemic. Various mutations have driven the rapid SARS-CoV-2 diversification, and some of them favor its better adaptation and circulation in humans, which may have determined the waxing and waning of various lineages.

## 1. Introduction

Although tremendous efforts have been taken to control corona virus disease 2019 (COVID-19) globally, it has still become an epidemic disease due to the constant appearances of novel variants and reinfection in humans. Considering the inevitability of novel zoonotic diseases in the future due to considerable diversity of viruses in wild animals and ongoing changes in ecology, environment, and social behavior worldwide (Shi et al. 2016; Chen et al. 2022, 2023; Holmes 2022), it is of great significance to better understand the evolutionary trajectory of severe acute respiratory syndrome coronavirus 2 (SARS-CoV-2) at the beginning of the COVID-19 outbreak for mitigating or preventing the damages or even disasters by future diseases.

SARS-CoV-2 viruses were initially divided into two lineages (termed A and B or S and L, respectively) (Rambaut et al. 2020; Tang et al. 2020). As the lineage A viruses share two identical nucleotides at sites 8,782 and 28,144 with bat-CoVs (RaTG13 and RmYN02), they were considered to emerge earlier in humans, even though they were identified relatively later than lineage B (Forster et al. 2020; Rambaut et al. 2020; Tang et al. 2020; Kumar et al. 2021; Pipes et al. 2021). Notably, one study proposed that SARS-CoV-2 emerged in humans via two or more separate spillover events (Pekar et al. 2022). The discoveries of close relatives of SARS-CoV-2 in bats from China and Southeast Asia indicate a bat origin of SARS-CoV-2 (Wu et al. 2020; Zhou et al. 2020; Temmam et al. 2022). In addition, an intermediate host may have been involved in this spillover event (Lu et al.2020b). However, the animal like civets and camels, which serve as intermediate hosts for severe acute syndrome coronavirus (SARS-CoV) and Middle East Respiratory syndrome coronavirus (MERS-CoV), respectively (Guan et al. 2003; Azhar et al. 2014), has not been identified so far. Therefore, it remains unclear when, where, and how SARS-CoV-2 first appeared in humans prior to its initial identification in December 2019 in Wuhan.

Various mutations appear when zoonotic viruses jump into and subsequently circulate in humans from their natural reservoir hosts (Duffy 2018). The mutations, which increase viral fitness and favor viruses to adapt to changing environments, will become fixed in new hosts (Domingo and Holland 1997). Compared to SARS-CoV and MERS-CoV, SARS-CoV-2 seems to adapt more rapidly and better in humans. Beneficial mutations, which facilitate the reproduction and transmission of SARS-CoV-2, were indeed identified (Rochman et al. 2021; Tao et al. 2021; Wu et al.2021a; Kistler, Huddleston, and Bedford 2022). Especially, the emergence of some mutations (e.g. A23403G, S: D614G) accelerated the global spread of lineage B (Korber et al. 2020; Plante et al. 2021; Volz et al. 2021). In contrast, lineage A eventually went extinct and replaced by lineage B. No matter how SARS-CoV-2 jumped into humans, the reasons are worth exploring.

Shanghai is an important transportation hub in and outside of China. The first COVID-19 case in Shanghai was reported on 20 January 2020. Since then, Shanghai Public Health Clinical Center as a designated hospital had received all adult COVID-19 cases appeared locally or imported from abroad until the beginning of 2022. Here, we recruited nearly all COVID-19 patients admitted to the hospital prior to October 2020, and performed epidemiologic investigations and phylogenetic analyses of genome sequences sampled from these patients as well as those sampled earlier and contemporaneously in and outside of China. Our data provide new insights on the evolutionary trajectory of SARS-CoV-2 at the beginning of the pandemic.

## 2. Materials and methods

### 2.1 Study design, patient cohort, and sample collection

Since the first COVID-19 patient was identified in Shanghai on 20 January 2020, who was from Wuhan, all adult clinically diagnosed and laboratory-confirmed patients in Shanghai were hospitalized at Shanghai Public Health Clinical Center during January 2020 to the beginning of 2022, including domestic and imported cases. We recruited nearly all cases (*n* = 933) admitted to the hospital during 20 January to 17 September 2020. The demographic, epidemiological, and clinical data of these patients were recorded by professional medical staffs.

Clinical samples including feces, anal swabs, throat swabs, sputum, nose swabs, and urine were collected by medical staffs as a part of routine clinical tests. Total RNA was extracted from these samples using RNeasy Plus Universal Mini Kit (Qiagen, Cat no. 73,404) following the manufacturer’s instructions. Then RT-qPCR was performed to detect SARS-CoV-2 according to the standard method provided by the China Center for Disease Control and Prevention by using the Bio Digital General qPCR kit (Jiangsu Saint Genomics, Cat no. CSJ-3-0018) following the product manual. The study was reviewed and approved by the ethics committee of Shanghai Public Health Clinical Center (YJ-2020-S018-02), together with the written informed consent from each patient.

### 2.2 Whole-genome amplification of SARS-CoV-2 by RT-nested PCR

In this study, SARS-CoV-2 genomes were recovered by two approaches, RT-nested PCR and next-generation sequencing. At the beginning, the full genomes were recovered from 199 patients (SH-P1 to SH-P199) using RT-nested PCR according to the early-established protocol through fifty-two pairs of primers designed based on the reference genome sequence Wuhan-Hu-1 (Supplementary Table S9) (Wu et al. 2020). In addition, 5ʹ- and 3ʹ-terminal regions of these genomes were determined using the Takara SMARTer RACE 5′/3′ kit (TaKaRa) following the manufacturer’s instructions. All PCR products were first detected by agarose gel electrophoresis and were sequenced by Sanger sequencing (Shanghai Sheng Gong Biotechnology Co., Ltd).

The sequencing results were assembled by SeqMan program of DNASTAR7.1 and then were compared to the reference sequence of lineage B (Wuhan-Hu-1, MN908947.3) or lineage A (Wuhan/WH04/2020, EPI_ISL_406801) by Nextclade (v2.9.1) (https://clades.nextstrain.org) (Aksamentov et al. 2021). Importantly, considering the fidelity of amplification, all nucleotide substitutions and deletions were double confirmed by the same methods.

### 2.3 Next-generation sequencing of the SARS-CoV-2 genome

To recover more genomes from patients with significant epidemiological history (SH-P200 to SH-P276), as well as to confirm the genomes, which occupied critical phylogenetic positions (lineage B0: SH-P37-2-Shanghai, SH-P55-2-Other-province-of-China-Henan; lineage A0: SH-P204-2-Wuhan, SH-P215-2-Shanghai), SARS-CoV-2 amplicon-based next-generation sequencing was performed. Briefly, the SARS-CoV-2 amplicon libraries were prepared using the Illumina COVID-Seq RUO Kits (Illumina) according to the manufacturer’s instructions and the v3 or v4 of the ARTIC COVID-19 multiplex PCR primers (https://artic.network/ncov-2019). The quantification of libraries was performed using a Qubit Flex fluorometer (Invitrogen), and libraries quality was assessed by the Qsep100 (Bioptic). Finally, paired-end (150-bp reads) sequencing of these libraries was performed on the MiniSeq Sequencing System (Illumina) at our laboratory.

After demultiplexing, the fastq files containing the raw reads were checked for quality criteria using FastQC (v.0.11.5) (http://www.bioinformatics.babraham.ac.uk/projects/fastqc/), and adapter sequences and low-quality end bases were trimmed using Trimmomatic (v.0.39) (Bolger, Lohse, and Usadel 2014). All clean reads were mapped to the SARS-CoV-2 genome (Wuhan-Hu-1, MN908947.3) using the BWA-MEM software package (version 0.7.17) (Li and Durbin 2010) with default parameters, and only uniquely best mapped reads were retained. In addition, primer sequences were removed from the aligned Binary Alignment/Map files using iVar (version 1.3.1) (Grubaugh et al. 2019). For SARS-CoV-2 genome assembly, only the sites, that had (1) a depth >10 reads, (2) a base quality score >30, (3) a mapping quality score >30, and (4) a consensus nucleotide identity > 80 per cent, were considered. All the remaining sites were masked with an ambiguous ‘N’. Finally, the genomes with epidemiological significance recovered using next-generation sequencing were further confirmed through RT-nested PCR.

### 2.4 SARS-CoV-2 genome data collection and lineage assignment

A total of 230,820 high-coverage SARS-CoV-2 viral genomes, which had definite sampling dates earlier than 30 September 2020, were retrieved from National Center for Biotechnology Information (NCBI) (https://www.ncbi.nlm.nih.gov/) or global initiative on sharing all influenza data (GISAID) database (https://www.gisaid.org/) (Supplementary Table S3). All the genome sequences matched the following criteria: (1) had definite sampling date and submission date within 1 year after the sampling date, (2) the length of genome sequences was longer than 29,000 nucleotides, (3) the number of ambiguous bases (N) was no more than 1,000, (4) the host was human, and (5) the ‘Passage details/history’ was not cell line or ‘unknown’. Finally, the genomes sampled before 29 February 2020, which had more than twenty nucleotide substitutions relative to Wuhan-Hu-1 (MN908947.3), were excluded.

### 2.5 Phylogenetic analysis of SARS-CoV-2

All SARS-CoV-2 genome sequences and outgroup genomes were first aligned using the FFT-NS-2 algorithm implemented in MAFFT (version 7) (Katoh and Standley 2013). For fidelity, two fragments at genomic positions 1–122 and 29,770–29,903 were excluded. The maximum-likelihood phylogeny of SARS-CoV-2 was estimated in IQ-TREE2 (Minh et al. 2020) using the general time-reversible nucleotide substitution model and empirical nucleotide frequencies.

Bayesian phylogenetic analysis was performed using the Hasegawa-Kishino-Yano substitution model with gamma-distributed rate variation and a strict molecular clock in Bayesian evolutionary analysis by sampling trees (BEAST, v.1.10.4) (Suchard et al. 2018). Bayesian analysis was run using Broad-platform evolutionary analysis general likelihood evaluator (Ayres et al. 2012) for a length of 50 million Markov chain Monte Carlo steps with a parametric exponential growth tree prior and sampled every 2,000 states. Four independent chains were run by the abovementioned methods and were subsequently combined by LogCombiner after removing 10 per cent of states as burn-in. The convergence was assessed with Tracer (v.1.7.2) (Rambaut et al. 2018). Maximum clade credibility summary trees were generated using TreeAnnotator (v.1.10.4). All trees were visualized using FigTree (v1.4.3) (http://tree.bio.ed.ac.uk/software/figtree/).

### 2.6 Lineage assignment of SARS-CoV-2

The definition of the SARS-CoV-2 lineages was based on the Pango Nomenclature (Rambaut et al. 2020) with few modifications. All SARS-CoV-2 genome sequences used in this study were first analyzed through Nextclade (v2.9.1) (https://clades.nextstrain.org) (Aksamentov et al. 2021). Based on the lineage assignment by Nextclade, nucleotide substitutions, and phylogenetic positions, viral genomes were defined as the following.

According to the nucleotides at the sites 8,782 and 28,144, the SARS-CoV-2 genomes recovered here and those retrieved from database were assigned as lineage A (8,782 T, 28,144C), lineage B (8,782C, 28,144 T), haplotypes ‘CC’ (8,782C, 28,144C), and ‘TT’ (8,782 T, 28,144 T). Within lineage A, the genomes that harbored a base ‘T’ at position 29,095 and clustered with the genome Guangdong/HKU-SZ-002/2020 (EPI_ISL_406030) (Chan et al. 2020) in the phylogenetic tree were defined as lineage A0. Within lineage B viruses, lineage B.1 and lineage B.4 were assigned according to Nextclade (v2.9.1). The genomes, which were recovered during stages 0 and I and located at the transition position between lineages B and B.1, were defined as lineage B–B.1. Similarly, the haplotype ‘TT’ genomes, which recovered during stage I and located at the intermediate position between lineage A and lineage B, were defined as lineage B0. Finally, the remaining genomes belonging to haplotype ‘TT’ or ‘CC’ were not included in further analysis unless they were clustered with lineage A or lineage B in the phylogenetic trees.

### 2.7 Estimation of the progenitor genome of SARS-CoV-2

To estimate the progenitor genome of SARS-CoV-2, bat-CoVs and pangolin-CoVs, which shared more than 85 per cent similarity to the reference genome Wuhan-Hu-1, were included (Supplementary Table S5). They were aligned using the FFT-NS-2 algorithm implemented in MAFFT (version 7) (Katoh and Standley 2013). For simplicity, all nucleotide positions referenced in this study were numbered according to Wuhan-Hu-1, and all the insertions relative to Wuhan-Hu-1 were ignored. In addition, according to the recombination breakpoints analyzed by Temmam and colleagues (Temmam et al. 2022), fifteen non-recombinant regions of bat-CoVs and pangolin-CoVs were included, but the regions exhibited less than 75 per cent similarity with SARS-CoV-2 were masked. For bat-CoVs, the nucleotide site, which had a dominant allele frequency higher than 75 per cent, was masked by the dominant allele artificially. Finally, a putative genome of bat-CoVs was estimated based on the dominant allele of each site and used for further comparison with SARS-CoV-2 and pangolin-CoVs (Supplementary Table S6).

### 2.8 Analysis of SARS-CoV-2 mutational landscape

For the comparison between bat-CoVs, pangolin-CoVs, and SARS-CoV-2, the terminal regions (1–236 and 29,726–29,903) and 1,585 bat-CoVs-highly polymorphic sites were excluded. Then, all sites were divided into six categories (category I to category VI). Among each lineage of SARS-CoV-2, the sites, which had a substitution frequency greater than 2 per cent and occurred more than once in any stage, were considered as SARS-CoV-2 polymorphic sites and were assigned as category V. The remaining sites were assigned into other five categories according to the variations in SARS-CoV-2 and pangolin-CoVs compared with the putative progenitor virus.

### 2.9 Statistical analysis

Categorical variables were analyzed by chi-squared test or fisher’s exact test. Non-normally distributed continuous variables were represented by median (with quartile range) or mean (with standard deviation). A two-sided Mann–Whitney U-test was used for two independent samples comparison, while the Kruskal–Wallis test was performed for multiple comparison test. All statistical analyses were performed using R (v.4.2.0) (https://www.r-project.org). A P-value of <0.05 was considered to be statistically significant.

## 3. Results

### 3.1 COVID-19 cases and their epidemiology

We recruited nearly all clinically diagnosed and laboratory-confirmed COVID-19 patients admitted to the Shanghai Public Health Clinical Center during 20 January to 17 September 2020, including the first case (a male) who was diagnosed in Shanghai on 20 January 2020 and from Wuhan (Supplementary Table S1). These patients included 553 males and 380 females, with a median age of 36.00 years.

**Figure 1.**
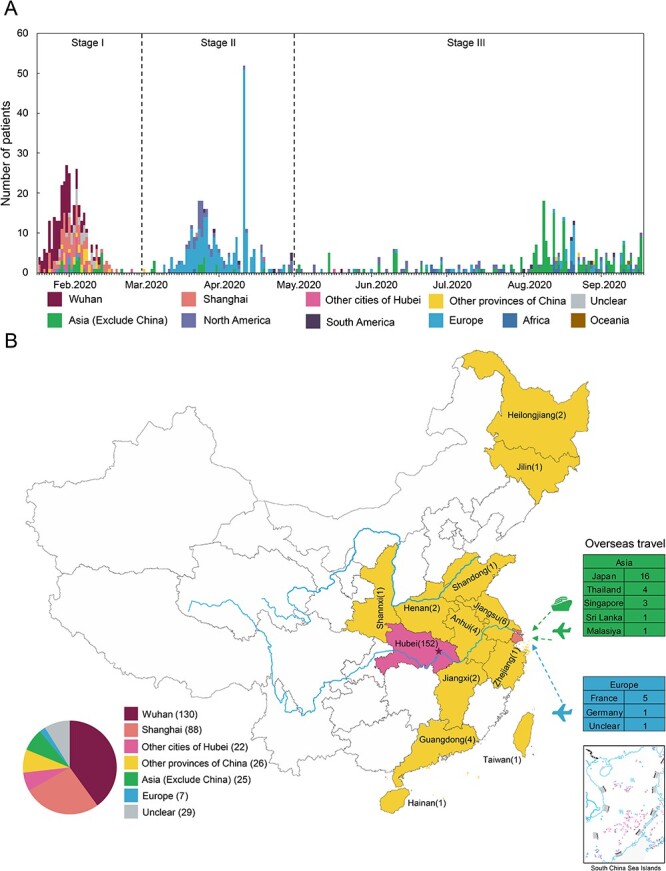
The COVID-19 cases and their epidemiological information in this study. (A) Timeline of the 933 COVID-19 cases we recruited in Shanghai Public Health Clinical Center before 17 September 2020. The cases were classified according to their geographic origins and represented by different colors. The detailed information of these patients can be found in Supplementary Table S1. According to the admission dates and origins of these patients, our study period was divided into three stages. (B) Geographic distribution of the 327 COVID-19 cases during stage I in this study. This map showed the location of the patients before they came to Shanghai (some patients always stayed in Shanghai) and were diagnosed with COVID-19.

Based on the admission dates of these patients, the whole study period was divided into three stages: stage I was from 20 January to 29 February 2020, stage II from 1 March to 30 April, and stage III from 1 May to 30 September (Fig. 1A). Of these 327 patients appeared during the stage I, 266 cases were infected domestically, 32 cases were diagnosed within 1–22 days after returning or coming from abroad, but the remaining 29 cases had no clear epidemiological records (Fig. 1B, Table 1). For domestic infections, 88 patients were infected in Shanghai, while 152 patients were from or had a travelling history to Wuhan (130) or other regions of Hubei province (22), and 26 patients were from or had a travelling history to other 12 Chinese provinces. During the stage II, 302 (99.01 per cent) cases were imported cases, who came or returned from abroad, mainly from Europe (231), while only three cases were infected domestically (Table 1, Supplementary Fig. S1). Similarly, during stage III, in addition to five domestical infections, 296 cases were infected outside of China, mainly from Asian countries and regions (Table 1, Supplementary Fig. S1). The epidemiological and clinical details were present in Supplementary Table S1.

**Table 1.** The demographical characteristics of patients among the three stages.

		Stage			
Characteristic	Total (*n* = 933)	Stage I (*n* = 327)	Stage II (*n* = 305)	Stage III (*n* = 301)	P value^$^
Age, median (IQR)	36.00 (26.00, 51.00)	51.00 (36.00, 64.00)	30.00 (21.00, 41.00)	32.00 (25.00, 44.00)	<0.001
Male, *n* (%)	553 (59.3)	168 (51.4)	182 (59.7)	203 (67.4)	<0.001
Geographic sources, *n* (%)					
China					
Wuhan	131 (14.0)	130 (39.9)	0 (0)	1 (0.3)	
Other cities of Hubei province	24 (2.6)	22 (6.7)	1 (0.3)	1 (0.3)	
Shanghai	89 (9.5)	88 (26.9)	1 (0.3)	0 (0)	
Other provinces of China	30 (3.2)	26 (8.0)	1 (0.3)	3 (1.0)	
Asia^$$^	210 (22.5)	25 (7.6)	17 (5.6)	168 (55.8)	
Europe	287 (30.8)	7 (2.1)	231 (75.7)	49 (16.3)	
Africa	30 (3.2)	0 (0.0)	1 (0.3)	29 (9.6)	
North America	90 (9.6)	0 (0.0)	47 (15.4)	43 (14.3)	
South America	12 (1.3)	0 (0.0)	6 (2.0)	6 (2.0)	
Oceania	1 (0.1)	0 (0.0)	0 (0.0)	1 (0.3)	
Unclear^$$$^	29 (3.1)	29 (8.9)	0 (0.0)	0 (0.0)	

$ P values for comparison of ages among three stages were calculated by Kruskal–Wallis test. P values for comparison of other variables were calculated by χ^2^ test or Fisher’s exact test. A two-sided of p value < 0.05 was considered to be statistically significant.

$ Except for China.

$$ No related information was recorded.

### 3.2 SARS-CoV-2 variants and their dynamics in Shanghai

Fecal samples and throat swab samples were collected from all these patients. A total of 343 complete or nearly complete SARS-CoV-2 genomes (>27,500 nucleotides in length) were recovered successfully from samples of 226 patients, including 177 genomes recovered from the samples collected serially from 60 patients (Supplementary Table S2). Finally, partial genomes ranging from 5,000 to 27,500 nucleotides were also obtained from other 41 patients.

**Figure 2.**
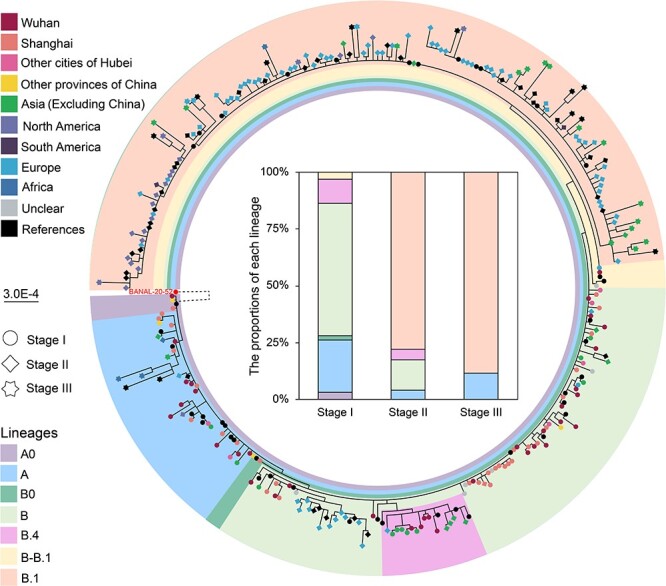
Phylogenetic analysis of SARS-CoV-2 genome sequences recovered from Shanghai and their dynamics. The maximum-likelihood phylogeny of SARS-CoV-2 genome sequences recovered in Shanghai and those with clear epidemiologic records sampled earlier and contemporaneously in and outside of China, with bat coronavirus BANAL-20-52 (MZ937000.1) as the outgroup. Only the earliest complete or near complete (>27,500 nucleotides) genome sequences recovered from each of patients were used here. Sequences recovered in this study were classified according to the stages and geographic sources of cases. According to the phylogenetic positions, these sequences were divided into seven lineages. The dynamics of SARS-CoV-2 lineages collected in this study during stages I–III were shown in the middle. Notably, the branch to the outgroup was not drawn to scale.

Based on the nomenclature described previously (Rambaut et al. 2020), these genomes fell into four main lineages (A, B, B.1, and B.4) in the phylogenetic tree estimated using the maximum-likelihood method (Fig. 2, Supplementary Fig. S2,  Supplementary Table S2). However, three genomes (SH-P204-2-Wuhan/29 January 2020, SH-P215-2-Shanghai/3 February 2020, and SH-P260-A-2-Anhui/7 February 2020) recovered here and one genome (Guangdong/HKU-SZ-002/2020) sampled from Guangdong on 10 January 2020 (Chan et al. 2020), formed a distinct lineage (here termed A0) and occupied the basal position of all SARS-CoV-2 variants. Compared to lineage A, lineage A0 shared an additional homologous nucleotide ‘T’ at site 29,095 with bat-CoVs. Notably, SH-P204-2-Wuhan/29 January 2020 was recovered from a patient who travelled to Shanghai from Wuhan on 9 January and admitted to the hospital on 23 January, SH-P215-2-Shanghai/3 February 2020 from a local individual having a close contact with a COVID-19 patient who arrived in Shanghai from Wuhan on 11 January, while SH-P260-A-2-Anhui/7 February 2020 was obtained from one, who travelled to Shanghai from Anhui province on 19 January and had a close contact with a COVID-19 case from Wuhan before arriving in Shanghai. The details of their epidemiologic data and sampling dates were provided in Supplementary Tables S1 and S2. Finally, five viral genomes recovered here and six genomes taken from GISAID were grouped into other two distinct lineages, which were termed as lineages B0 and B–B.1 due to their positions located between lineages A and B and between lineages B and B.1 in Fig. 2, and they occupied basal positions of lineages B and B.1, respectively. Hence, these data reveal the co-circulation of diverse SARS-CoV-2 variants in Shanghai at the beginning of the COVID-19 outbreak.

Overall, the phylogenetic positions of these viral genomes did not strictly follow the chronological order (Fig. 2). During the stage I, 56 infections including 51 domestic and five imported cases were caused by lineage B, while 22 infections including 20 domestic and two imported cases were associated with lineage A (Fig. 2). In addition, three, two, three, and 10 infections were associated with lineages A0, B0, B–B.1, and B.4, respectively. Notably, one patient infected by B–B.1 viruses was a female who returned from Germany on 23 January 2020. However, during the stage II, in addition to a few of cases associated with viruses from lineages B (*n* = 14), B.4 (*n* = 5), and A (*n* = 4), the majority of patients were caused by lineage B.1 (*n* = 81). During stage III, the majority of viruses recovered here belonged to lineage B.1 (*n* = 23). Although lineage A viruses were still present (*n* = 3), other variants were not found. Clearly, the dynamics of SARS-CoV-2 in Shanghai was similar to that in other Chinese parts and the world.

### 3.3 Phylogeny of SARS-CoV-2 variants at the beginning of the COVID-19 outbreak

**Figure 3.**
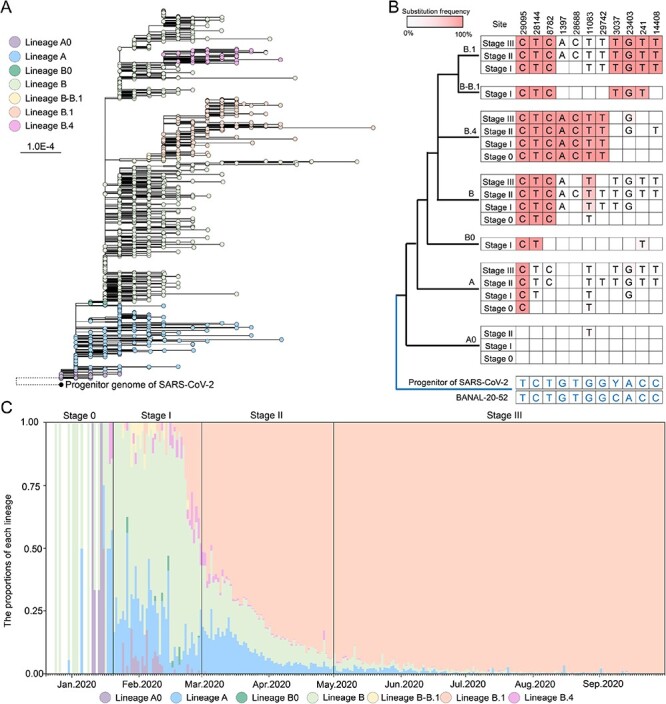
Phylogeny and mutational landscape of SARS-CoV-2 variants and their dynamic at the beginning of COVID-19 outbreak. (A) The maximum-likelihood tree of 1,704 SARS-CoV-2 genomes collected here (coverage >27,500) and retrieved from NCBI and GIASID (see ‘Materials and methods’ section) during stage 0 and stage I, with the progenitor genome of SARS-CoV-2 as outgroup. Here, we only displayed one scenario that lineage A0 was located at the root of SARS-CoV-2 phylogeny, while the other three possible scenarios were shown in Supplementary Fig. 4. Notably, the branch to the outgroup was not drawn to scale. (B) The mutation landscape of each lineage at eleven lineage-associated sites during stages 0–III. The schematic tree on the left illustrates the phylogenetic relationships between the progenitor and different lineages of SARS-CoV-2. The substitution frequencies were indicated by different shades of red. Empty squares indicate that no substitution occurred at that position in the corresponding lineage and stage. (C) The dynamic of lineages A0, A, B0, B, B.4, B–B.1, and B.1. The daily proportion of lineages was calculated based on the number of genomes collected for each lineage per day. Notably, due to the low number of sequences at the beginning of stage 0, the proportions plotted were likely quite noisy.

We further performed phylogenetic analysis of genomes recovered here during stage I as well as those collected in and outside of China during the same and earlier period (before 20 January 2020, here termed ‘stage 0’). Herein, a putative progenitor genome of SARS-CoV-2, which was reconstructed based on diversities of bat-CoV genomes (see ‘Materials and methods’ section), was used as an outgroup. Notably, the root position of SARS-CoV-2 phylogeny was uncertain, as approximately 30 per cent genomes within lineage A also harbored one substitution toward the outgroup relative to the reference genome of lineage A (Supplementary Fig. 3). Overall, there were four candidates for the root position when outgroup rooting was assumed: lineage A0 (A + 29,095 T) (Fig. 3A), lineage A + 18060 T, lineage A + 3171C, and lineage A + 24023 T (Supplementary Fig. S4). Considering that the A0 variants were identified in patients known to have been infected at least 9 days earlier than other three variants (Supplementary Fig. S5), particularly, the first A0 virus was recovered from a male who developed symptoms as early as 4 January 2020, after having an indirect contact with an unknown pneumonia patient hospitalized on 27 December 2019, in Wuhan (Chan et al. 2020), lineage A0 was the most likely candidate for the original lineage of SARS-CoV-2, although other possible roots were equally close to the outgroup as well (Fig. 3A).

Similarly, when the bat coronavirus BANAL-20-52 was used as an outgroup, the root position was also uncertain (Supplementary Fig. S6). Nevertheless, lineage A0 was still a reasonable choice for the original lineage (Supplementary Fig. S7). In addition, all A0 genomes still clustered together and formed a lineage distinct from lineage A in the phylogenetic tree. Combined with the mutational features of various lineages (see further), the lineage A0 viruses were more likely to emerge earliest in humans. Notably, two B0 genomes recovered here and seven genomes recovered from Wuhan, Singapore, and the UAE were at the basal position of lineage B and its descendants (abbreviated as Lineage Bs). The B–B.1 genomes recovered here clustered together with those sampled from Sichuan, Guangdong, Australia, and Germany, and they occupied the basal position of all B.1 viruses. Finally, the genomes from lineages A, B, B.4, and B.1 collected in and outside of Shanghai fell into their respective groups.

Next, we sought to depict the mutational signatures of these variants (Fig. 3B). Compared to bat virus BANAL-20-52 and the putative progenitor genome, eleven nucleotides in SARS-CoV-2 genomes, which served as signature marks of early lineages, underwent mutation during the early period, and some of them occurred successively. Notably, these signature marks did not appear in all lineage A0 genomes, with the exception of one genome (France/OCC-SC470/2020) sampled from France on 9 March, which had the mutation G11083T. However, compared to lineage A0, the mutation T29095C appeared in almost all viruses from lineages A, B0, and B. The mutation C28144T was not observed in all lineage A0 and the majority of lineage A genomes (99.68 per cent) but appeared in almost all genomes from lineages B0, B, and Bs. Interestingly, the mutation T8782C was not found in all lineages A0, B0, and the majority of lineage A viruses (98.57 per cent), but emerged in almost all B and Bs genomes. In addition, haplotype ‘CC’ or ‘TT’ also appeared in a few of viruses from lineage Bs, indicative of the regressive evolution at these sites, although some of them may be resulted from bioinformatics artifacts (Pekar et al. 2022). More lineage-related signature mutations were observed in lineage Bs genomes over time, especially in lineages B.1 and B.4 genomes. In sum, these data were consistent with that all known early-identified SARS-CoV-2 variants seem to have originated from one common ancestor, probably lineage A0, and kept on evolving in human populations.

Finally, we also analyzed the dynamics of these variants at the beginning of the outbreak (Fig. 3C). During stage 0, lineage B dominated the prevalence (*n* = 45, 68.18 per cent), with the proportion over that of lineage A by more than three times (*n* = 13, 19.70 per cent). Remarkably, the proportion of lineage A decreased rapidly over time and was gradually replaced by lineage Bs (Fig. 3C). During stage 0, apart from lineages A and B, lineage A0 genomes were identified from six patients in Guangdong province (Chan et al. 2020; Kang et al. 2020). Besides the first patient mentioned above, his wife and daughter also exhibited clinic signs on 3 and 4 January and 2020, respectively, after they visited a febrile pneumonia patient on 29 December 2019, in Wuhan, who hospitalized on 27 December 2019. However, lineage A0 became less and less (stage I, *n* = 36, 2.20 per cent; stage II, *n* = 13, 0.02 per cent), and finally disappeared during stage III. The two earliest lineage B.4 genomes were identified on 18 January in Wuhan and 19 January in Shandong province, respectively. Interestingly, apart from the signature substitutions of lineage B.4, they contained one (T11709A) and three additional substitutions (C7299T, C10789T, and C27612G), respectively, suggesting that the prototype strain of lineage B.4 appeared earlier. Together, all these data revealed the quick diversification of SARS-CoV-2 at the beginning of COVID-19 outbreak, with the lineage Bs becoming dominant gradually.

### 3.4 The evolutionary routes of lineage B.1

**Figure 4.**
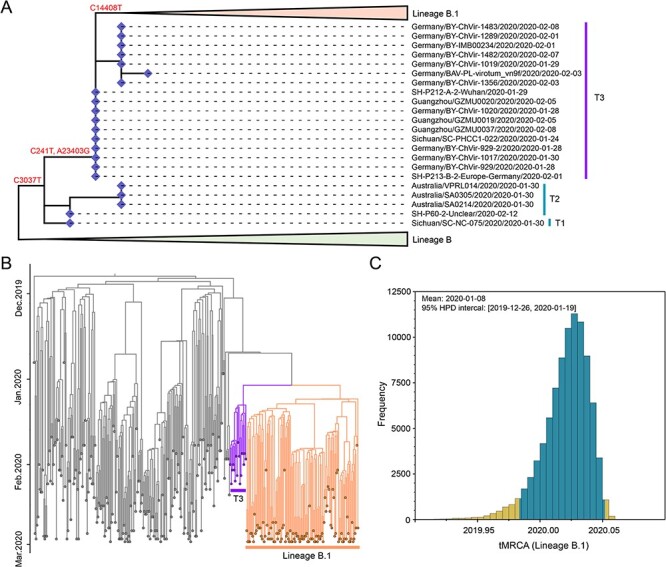
The evolution trajectory of lineage B.1. (A) The detail phylogeny of lineages B, B–B.1, and B.1. This is a magnification of phylogenetic tree of Fig. 3A, with all genomes except for lineage B–B.1 were collapsed. (B) Time-scaled Bayesian phylogenetic analysis of 336 representative SARS-CoV-2 genomes. Sequences in lineage B.1 and the group T3 were marked with different colors. (C) Estimates of the time of the most recent common ancestor (tMRCA) of lineage B.1.

Although lineage B.4 was identified earlier than lineage B.1, the emergence of lineage B.1 was a pivotal event for the global pandemic (Fig. 3C). To explore the evolutionary routes of lineage B.1, we performed phylogenetic analysis of lineages B, B–B.1, and B.1 genomes sampled during stages 0 and I. Clearly, lineage B–B.1, which was comprised of the genomes recovered in Australia, China, and Germany, located at the transition position between lineages B and B.1. This lineage characterized with the mutation C3037T was separated into three sub-groups (T1, T2, and T3) (Fig. 4A). In addition to the mutation C3037T, T1 and T2 genomes, which were sampled during 30 January to 12 February, contained mutations C16247T and C27213T, respectively, while T3 sampled during 24 January to 15 February carried other two mutations (C241T and A23403G). Finally, although lineage B–B.1 was reported in Sichuan province as early as 24 January, the theoretical ancestor, which only carried the mutation C3037T, was not found.

Lineage B.1 genomes were first identified in Australia on 25 January, and then in UK and Saudi Arabia on 3 February, as well as in more countries 3 weeks later. However, based on the time-calibrated phylogeny by Bayesian inference, the emergence of the most recent common ancestor of lineage B.1 was dated to 8 January 2020 (95 per cent HPD: 26 December 2019, to 19 January 2020; Fig. 4B and C), which was prior to the identification of all identified B-B.1 viruses. Notably, besides the lineages B–B.1 and B.1, the lineage B.1-related mutations (C241T, C3037T, C14408T, and A23403G) also emerged sporadically in both lineage A and other sub-lineages of B during stages I–III, although we cannot exclude the possibility that some of them may be resulted from bioinformatics errors (Supplementary Table S4).

### 3.5 Mutational landscape of SARS-CoV-2 compared to coronaviruses in bats and pangolins

**Figure 5.**
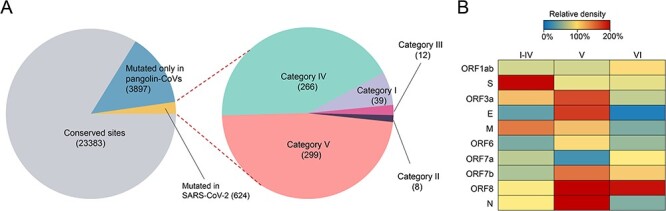
Mutational landscape of SARS-CoV-2 compared to coronaviruses in bats and pangolins. (A) The comparison and classification of 27,904 sites among coronaviruses from three hosts. All the sites were classified according to the consistencies or differences of the nucleotides among SARS-CoV-2, pangolin-CoVs, and bat-CoVs. (B) The relative density of nucleotide sites in different categories across each gene of SARS-CoV-2. We set the density of sites that evenly distributed across the genome to 100 per cent.

Finally, we attempted to explore the mutational landscape of SARS-CoV-2 in humans at the early period of COVID-19 outbreak through the comparison of the mutations appeared in all available SARS-CoV-2 genomes (*n* = 231,163) sampled here and those in and outside of China during stages 0–III, as well as SARS-CoV-2-related viral genomes (*n* = 8) from pangolins which sharing >85 per cent similarity with SARS-CoV-2 and the putative progenitor genome (Supplementary Tables S5 and S6). Consequently, a total of 27,904 nucleotide sites were included in our analyses, while the remaining 1,585 sites were excluded due to their high polymorphism within bat-CoVs. Relatively to the putative progenitor genome, nucleotides at 23,383 (83.80 per cent) sites were conserved, but mutation occurred at 4,521 (16.20 per cent) sites of SARS-CoV-2 or pangolin-CoV genomes (Fig. 5A). Based on nucleotide variations and genomes’ origins, these mutations could be divided into six categories: (1) mutation at each of thirty-nine sites was identical for all SARS-CoV-2 and pangolin-CoVs; (2) mutation at each of eight sites was identical for all SARS-CoV-2 or all pangolin-CoVs, but different between SARS-CoV-2 and pangolin-CoVs; (3) mutation at each of twelve sites was identical in all SARS-CoV-2 and some of pangolin-CoVs, but different in remining pangolin-CoVs; (4) mutation at each of 266 sites was identical in all SARS-CoV-2, but mutation did not occurred in most of these sites (170/266) of pangolin-CoVs; (5) mutation at each of 299 sites occurred in all or some of SARS-CoV-2, and a few of pangolin-CoVs, but mutation was irregular; and (6) mutation at each of 3,897 sites appeared only in pangolin-CoVs (Fig. 5A, Supplementary Table S6). Notably, the first four categories of mutations mainly appeared in spike gene, but category V mutations enriched in both ORF8 and N genes and category VI mutations mainly in ORF8 gene (Fig. 5B). These data suggest that some of categories I–IV mutations may be necessary for the finally successful jumping of progenitor(s) of SARS-CoV-2 into humans from bats, and some of them are beneficial for the ongoing spread of SARS-CoV-2 in humans.

## 4. Discussion

The discoveries of diverse SARS-CoV-2-related coronaviruses in bats from China, Japan, and Southeast Asia suggest that bats are the natural reservoir host of SARS-CoV-2 (Hu et al. 2018; Murakami et al. 2020; Zhou et al. 2021; Temmam et al. 2022). However, even when SARS-CoV-2 is compared to the closest bat virus (BANAL-20-52), the difference between them reaches >900 nucleotides. Although SARS-CoV and MERS-CoV are also believed to have originated from bats (Li et al. 2005; Ithete et al. 2013), they jumped into humans via intermediate hosts civets and camels, respectively, with >99.8 per cent similarity to progenitor viruses identified in intermediate hosts (Guan et al. 2003; Azhar et al. 2014). To better understand SARS-CoV-2 mutational history, the genome of a hypothetical progenitor of SARS-CoV-2 was reconstructed by two research groups (Kumar et al. 2021; Pekar et al. 2022), with 99.98 per cent and 98.72 per cent similarity to the first-identified A0 virus (Guangdong/HKU-SZ-002/2020), respectively (Supplementary Table S7). Our reconstructed progenitor genome shared 98.79 per cent similarity to the A0 virus (Supplementary Table S7), which was similar to that reconstructed by Pekar and his colleagues (Pekar and Pekar et al. 2022). Notably, the substitutions at 59 sites (categories I–III) were also emerged in all known pangolin viruses. Hence, although the possibility of an undetected bat virus as the source of COVID-19 could not be ruled out, all known bat coronaviruses are unlikely the direct source for the epidemic, and an unidentified animal, which may serve as an intermediate host, could be responsible for the evolution and the final cross-species event of SARS-CoV-2.

Two lineages (A and B or L and S) of SARS-CoV-2 were identified in human populations at the beginning of the COVID-19 outbreak (Rambaut et al. 2020; Tang et al. 2020). Lineage A was considered to emerge in humans earlier than lineage B (Rambaut et al. 2020). However, this view contradicts the fact that lineage B was detected earlier and more frequently than lineage A (Pipes et al. 2021). The circulation of intermediate haplotypes ‘C/C’ or ‘T/T’ (here lineage B0) means the evolution of lineage A toward lineage B in humans. However, these intermediate haplotypes were considered to be likely resulted from artifacts of contamination or bioinformatics, or from lineage A via convergent evolution or reversion, hence, it was proposed that lineages A and B emerged in humans via separate introductions or SARS-CoV-2 emergence was resulted from multiple zoonotic events (Pekar et al. 2022).

The first-identified lineage A0 genome was from a patient in Guangdong on 10 January 2020 (Chan et al. 2020). Although lineage A0 belongs to T/C haplotype (8782 T and 28144C), they differ from lineage A viruses by the nucleotide ‘T’ at site 29095, which was also shared by all known bat and pangolin SARS-CoV-2-related coronaviruses. Herein, lineage A0 genomes were recovered from three patients in Shanghai, who were from Wuhan or had a close contact with confirmed cases from Wuhan. Compared to the first-recovered A0 genome, each of them has one additional mutation (C16624A, C23347T, and G25947T) different from bat coronaviruses. Of all the six earliest lineage A0 genomes recovered in Guangdong during the stage 0, five genomes were identical, but the last one contained two additional mutations. In addition, lineage A0 genomes with more mutations were also found in other eight Chinese provinces and other five countries during stages I and II, indicating the widespread and constant evolution of lineage A0 in human populations. Importantly, the first A0 case in Guangdong was related to a febrile pneumonia patient hospitalized in Wuhan on 27 December 2019 (Chan et al. 2020). Thus, the onset date of this patient infected with lineage A0 might not be later than that of the first known patient infected with lineage A (onset date: 26 December 2019) (Lu et al.2020b) or lineage B (onset date: 20 December 2019) (Wu et al. 2020). All these data suggest that lineage A0 virus was the most likely original form among all known early identified viruses in China. Considering that other three candidates were also equally close to the root and there were probably some early infections that were not sequenced, the possibility that a lineage is closer to the root than lineage A0 could not be excluded.

Lineage B0 viruses (haplotype ‘T/T’) were reported in Wuhan, Singapore, and the UAE (Supplementary Table S3) and occupied the intermediate position of lineage A and lineage B. Herein, lineage B0 genomes were recovered from two patients, who were infected at different regions (Henan and Shanghai) and hospitalized on 4 and 8 February, respectively. Interestingly, our genomes share 100 per cent similarity with four genomes sampled from Wuhan and Singapore. Overall, these genomes have no difference from the reference genome of lineage A or B with the exception of the nucleotide at the site 8,782 or 28,144, but it should be noted that there was an indeterminate C/A nucleotide assignment (1,125 counts of ‘C’ and 2,008 counts of ‘A’) at the position 27,230 of SH-P37-2-Shanghai genome. Finally, either lineage A0 or B0 genomes were recovered and confirmed by both RT-PCR and transcriptomic protocol (SRR25229357, SRR25229358, SRR25229360, and SRR25229361). Hence, these data indicate the true existence and evolution of lineages A0 and B0 viruses (Supplementary Table S8) in human populations.

The emergence of lineage B.1 was a key event during the COVID-19 pandemic (Korber et al. 2020; Plante et al. 2021; Volz et al. 2021). Interestingly, the haplotype (lineage B–B.1) intermediate to lineages B and B.1 was reported in two Chinese provinces (Guangdong and Sichuan), as well as Australia and Germany (Bohmer et al. 2020; Lu et al. 2020a). Herein, seven B–B.1 genomes from three cases (SH-P60, SH-P212, SH-P213) were also recovered. Compared to lineage B, the genome SH-P60-2-Unclear/12 February 2020 harbored mutations C3037T and C27213T, which were also observed in three genomes sampled from Australia on 30 January 2020. Like the B–B.1 genomes sampled from Sichuan and Guangdong provinces as well as Germany, genomes recovered from other two cases (SH-P212 and SH-P213) shared the mutations C241T, C3037T, and A23403G. In addition, lineage B.1-related mutations also appeared sporadically in other lineages during stage I, including mutation A23403G (S: D614G) occurred in ten genomes sampled from Wuhan and Shanghai, mutation C241T occurred in one genome sampled from the UAE, and mutation C3037T occurred in one genome sampled form Australia (Supplementary Table S4). Therefore, all these data reveal the appearance of diverse haplotypes during the evolution of lineage B.1 from B.

Generally, viruses undergo multiple mutations to better adapt to the new genetic environment when they spillover into a new host (Domingo and Holland 1997; Duffy 2018). Early studies also revealed the occurrence and fixation of low-frequency mutations within individual (Voloch et al. 2021; Manuto et al. 2022; Landis et al. 2023) and among individuals (Bohmer et al. 2020; Popa et al. 2020; Sekizuka et al. 2020; Chau et al. 2021). Herein, we also observed the intra-individual substitutions (Supplementary Table S2). The mutation rate of SARS-CoV-2 has been estimated as approximately two substitutions per genome per month for the early phase of the epidemic (Duchene et al. 2020; Brussow 2021; Pekar et al. 2021). Our phylogenetic and mutational landscape analyses also indicate the constant mutations during the early outbreak days (Fig. 3B). Although the differences among these lineages were initially subtle, considering the high transmissibility, it is more likely that the nucleotide changes observed in lineages A0, A, B0, and B arose during the ‘cryptic transmission period’ of SARS-CoV-2, which spanned from the identification of the first COVID-19 infection or even earlier, to the date of the first identification of the SARS-CoV-2 genome Wuhan-Hu-1. Finally, more sub-lineages were subsequently diversified from lineage A or B, including the well-known lineages B.1 and B.4. Therefore, all known SARS-CoV-2 viruses including A0, A, B0, and B seem to be from a common progenitor virus, which might have jumped into humans via a single spillover event, rather than two or multiple zoonotic events (Pekar et al. 2022). Their co-circulation at the early phase of the epidemic might have resulted from rapid evolution of SARS-CoV-2 in human populations worldwide.

Although the evolution of SARS-CoV-2 is characterized primarily by purifying selection, a small set of sites including the spike and nucleocapsid protein, especially the mutations which emerged independently and parallelly with a high frequency in multiple lineages, appeared to evolve under positive selection (Rochman et al. 2021; Kistler, Huddleston, and Bedford 2022). Among them, beneficial mutations, including D614G and N501Y in spike gene and R203K/G204R in nucleocapsid gene, have been found to increase SARS-CoV-2 fitness and transmissibility in human populations (Korber et al. 2020; Plante et al. 2021; Volz et al. 2021; Wu et al.2021b; Liu et al. 2022). Similarly, the adaptive evolution was also observed during the onward transmission of SARS-CoV-2 in animals after human-to-animal spillover (Lu et al. 2021; Oude Munnink et al. 2021; Hale et al. 2022; Tan et al. 2022). Despite the appearance and co-circulation of several lineages during the early phase of the epidemic, only lineage B and its descendants (especially B.1) dominated the later epidemic. Epistatic effect was observed during the SARS-CoV-2 evolution (Zeng et al. 2020; Rochman et al. 2021; Starr et al. 2022), which may explain somewhat why four crucial B.1-related mutations appeared first in lineage B. In contract, although the B.1-related mutations also appeared in lineage A and had >10 per cent prevalent rate during stage III, their low prevalence and various intervention measures toward COVID-19 may have led to their replacement by lineages B and Bs. Finally, the founder effect may also have shaped the proportions among lineages, just as observed when COVID-19 introduced into Spain and Canada (Diez-Fuertes et al. 2021; Murall et al. 2021).

In sum, although multiple lineages of SARS-CoV-2 were co-circulating during the early period of the COVID-19 epidemic, they still exhibited the evolutionary continuity. All of them may have evolved from one common ancestor, probably lineage A0 or an unidentified close relative, and jumped into human via a single zoonotic event. Various mutations have driven the rapid diversification of SARS-CoV-2, with some being beneficial for its better adaptation and circulation in humans, which may have determined the waxing and waning of various lineages.

## Supplementary Material

veae020_Supp

## Data Availability

All virus sequences generated in this study have been deposited in GenBank under the accession numbers OR240356-OR240723 and OR251846-OR251847. The SARS-CoV-2 amplicon-based next-generation sequencing reads generated in this study are available at the NCBI Sequence Read Archive (SRA) database under the BioProject accession PRJNA993399.
