# Supplementary material for: Evolutionary trajectory of diverse SARS-CoV-2 variants at the beginning of COVID-19 outbreak
Source: Virus Evol. 2024 Mar 5;10(1):veae020. doi: 10.1093/ve/veae020 (PMC10984623; doi:10.1093/ve/veae020)
Supplement: veae020_Supp [file veae020_supp.zip › suppl_data/Supplementary Figure legends.docx]

Supplementary Materials for

**Evolutionary trajectory of diverse SARS-CoV-2 variants at the beginning of COVID-19 outbreak.**

Jia-Xin Lv, Xiang Liu, Yuan-Yuan Pei, Zhi-Gang Song, Xiao Chen, Shu-Jian Hu, Jia-Lei She, Yi Liu, Yan-Mei Chen, Yong-Zhen Zhang*

*Corresponding author Email: zhangyongzhen@fudan.edu.cn

**This file includes:**

Supplementary Figure legends 1 to 7

**Other Supplementary Material for this manuscript includes the following**:

Supplementary Tables 1 to 9

**Supplementary Fig. 1. The Geographic distribution of the 598 imported COVID-19 cases and eight native cases during stages II and III.** The origin countries of these cases were marked with corresponding colors on the map according to their continents. Countries that had more than five cases were additionally labeled with text.

**Supplementary Fig. 2. The maximum-likelihood phylogeny of SARS-CoV-2 genome sequences from Shanghai.** The tree was a detailed version of Fig. 2. SARS-CoV-2 genome sequences recovered in this study were classified according to the stages and geographic sources of cases. Notably, the branch to the outgroup was not drawn to scale.

**Supplementary Fig. 3. The uncertain root position of SARS-CoV-2 when the** **putative progenitor genome of SARS-CoV-2 was used as an outgroup.** The tree only contained the viral genomes from lineages A and A0. Genomes harbored substitutions toward the outgroup relative to the reference sequence of lineage A were displayed by a red diamond, and their phylogenetic diagram, collection time, and substitutions relative to the lineage A were shown. Substitutions toward the outgroup were marked in red. Candidates for the root position of SARS-CoV-2 phylogeny were marked with a star, and the four corresponding trees were shown in Fig. 3A and Supplementary Fig. 4.

**Supplementary Fig. 4. Three possible maximum-likelihood trees of 1704 SARS-CoV-2 genomes collected during stage 0 and stage I with the progenitor genome of SARS-CoV-2 as outgroup.** Here, we presented other three possible scenarios of the root position of SARS-CoV-2 phylogeny apart from lineage A0 (See Fig. 3A). Notably, the branch to the outgroup was not drawn to scale.

**Supplementary Fig. 5. The dates of genome collection and epidemiology of patients associated with the four candidate root lineages.** The number of genomes collected for each lineage and significant events have been clearly arranged on a timeline, different colored bars represent distinct lineages. For lineage A+18060T, there were 17 and 25 genomes recovered on February 28th and 29th, respectively, which are distinguished by bottle green. For lineage A+24023T, no detailed epidemiological information was available.

**Supplementary Fig. 6. The uncertain root position of SARS-CoV-2 when the** **bat-CoVs BANAL-20-52 (MZ937000.1) was used as an outgroup.** The tree only contained the viral genomes from lineages A and A0. Genomes harbored substitutions toward the outgroup relative to the reference sequence of lineage A were displayed by a red diamond, and their phylogenetic diagram, collection time, and substitutions relative to the lineage A were shown. Substitutions toward the outgroup were marked in red. Candidates for the root position of SARS-CoV-2 phylogeny were marked with a star, and the two corresponding trees were shown in Supplementary Fig. 7.

**Supplementary Fig. 7. Two possible maximum-likelihood trees of 1704 SARS-CoV-2 genomes collected during stage 0 and stage I with the bat-CoVs BANAL-20-52 as outgroup.** Here, we presented other two possible scenarios of the root position of SARS-CoV-2 phylogeny (left: lineage A0, right: lineage A+24023T). Notably, the branch to the outgroup was not drawn to scale.
