# Supplementary figures and images for: Evolutionary trajectory of diverse SARS-CoV-2 variants at the beginning of COVID-19 outbreak
Source: Virus Evol. 2024 Mar 5;10(1):veae020. doi: 10.1093/ve/veae020 (PMC10984623; doi:10.1093/ve/veae020)

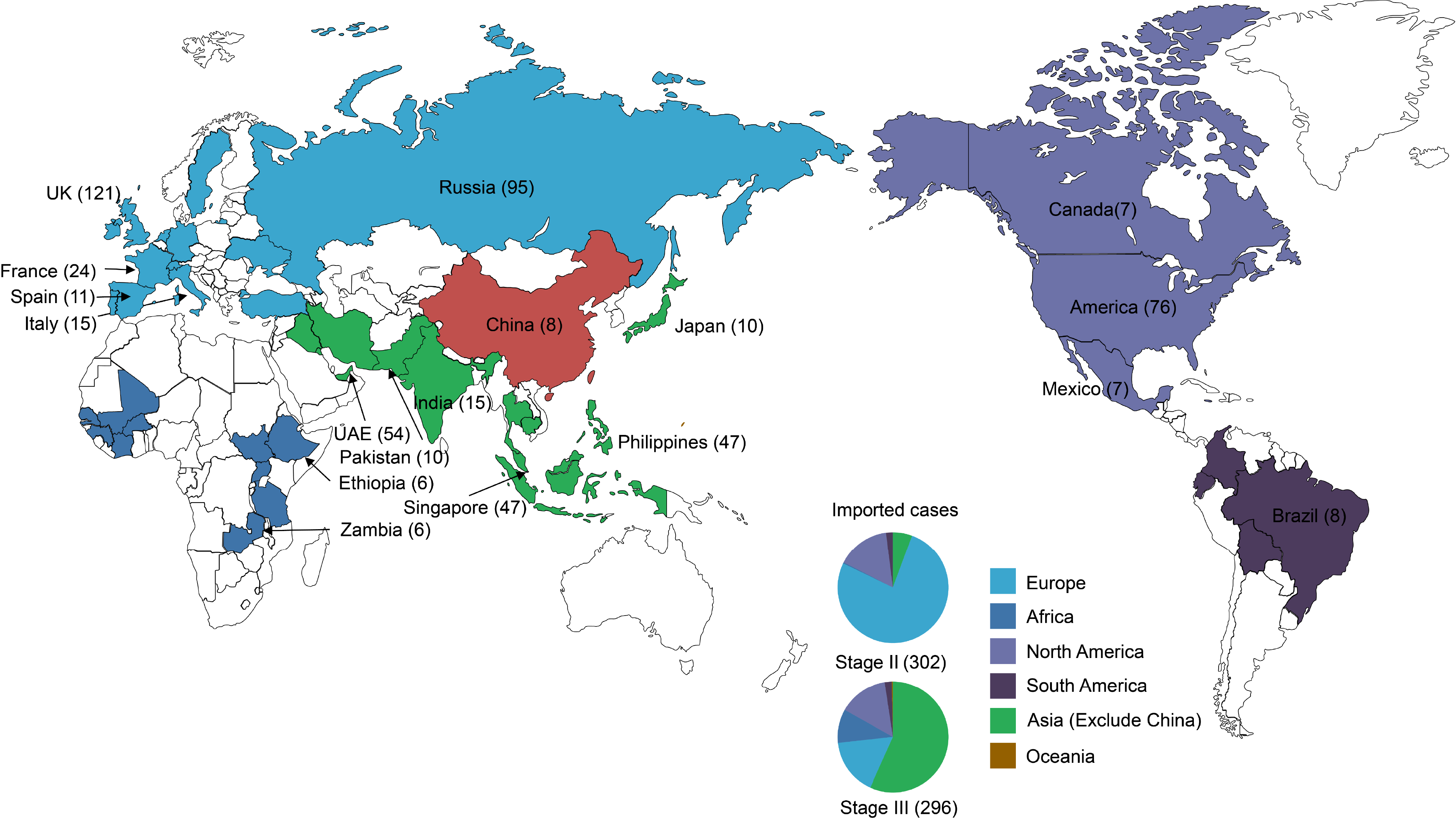

Supplement: veae020_Supp [file veae020_supp.zip › suppl_data/Supplementary Fig. 1.png]

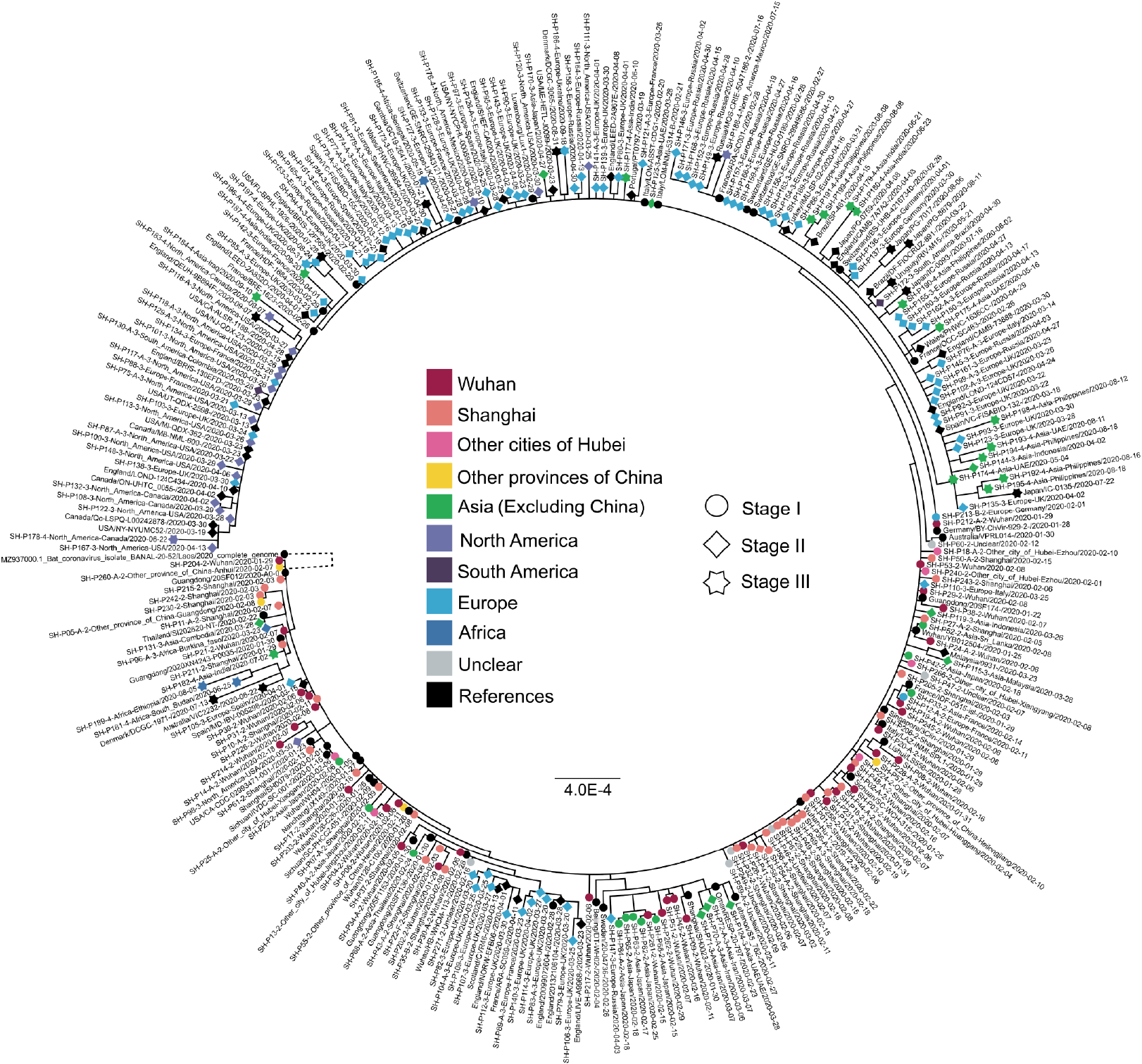

Supplement: veae020_Supp [file veae020_supp.zip › suppl_data/Supplementary Fig. 2.png]

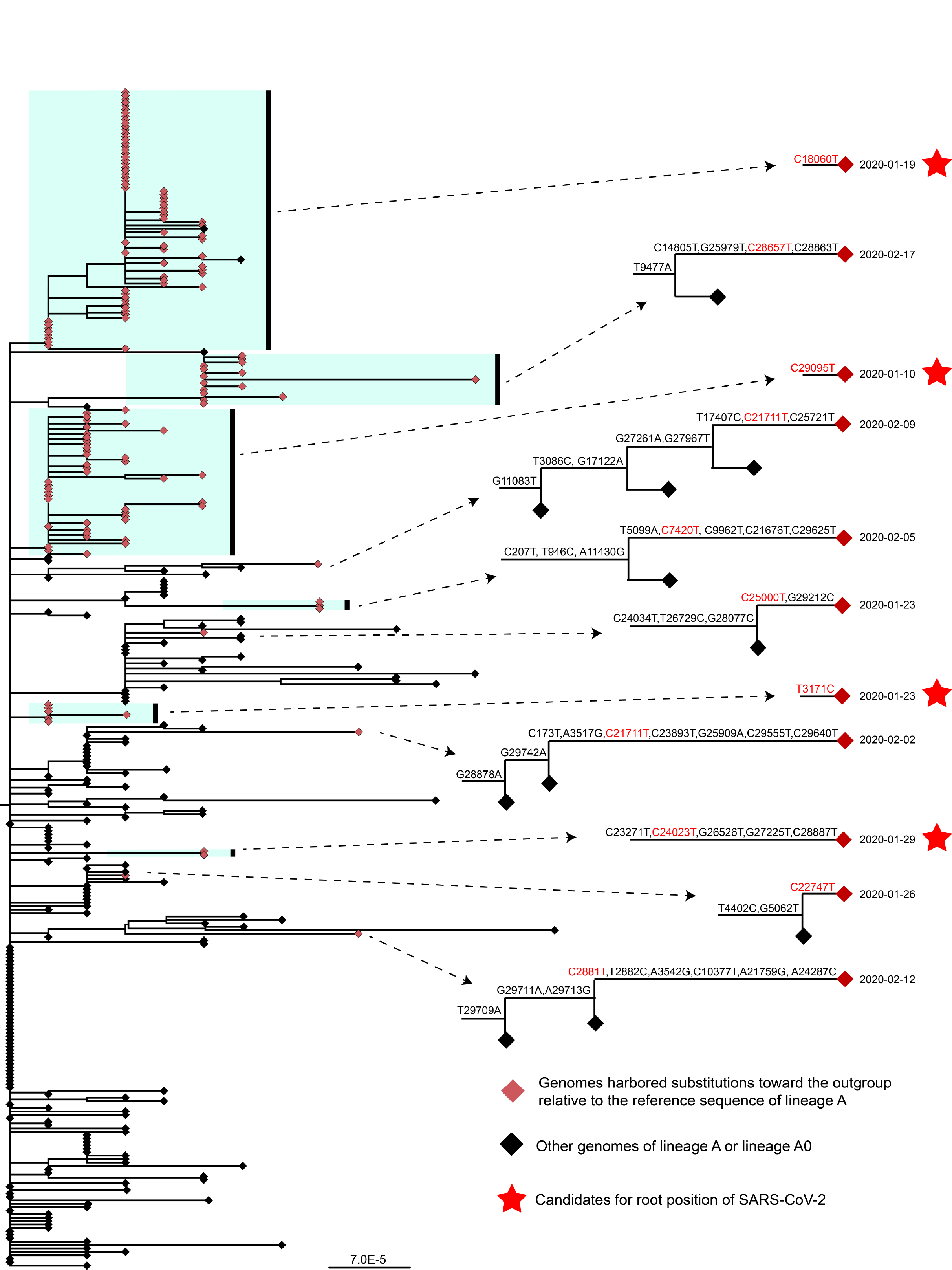

Supplement: veae020_Supp [file veae020_supp.zip › suppl_data/Supplementary Fig. 3.png]

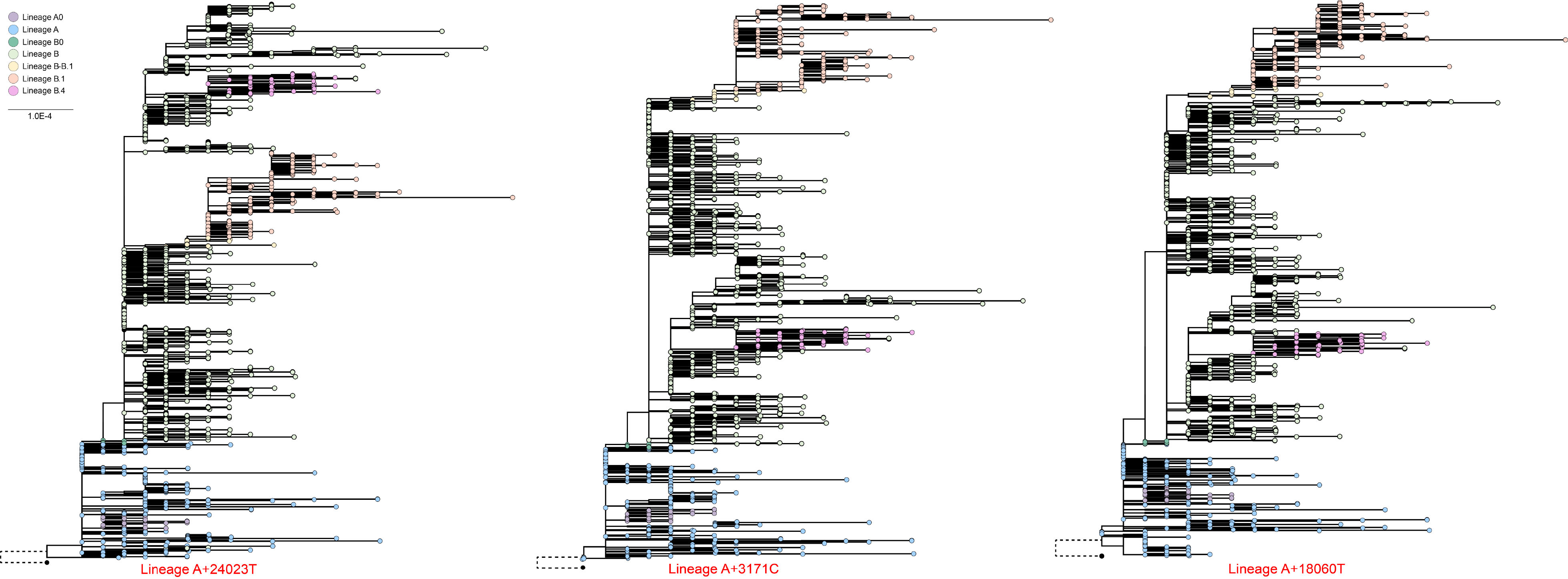

Supplement: veae020_Supp [file veae020_supp.zip › suppl_data/Supplementary Fig. 4.png]

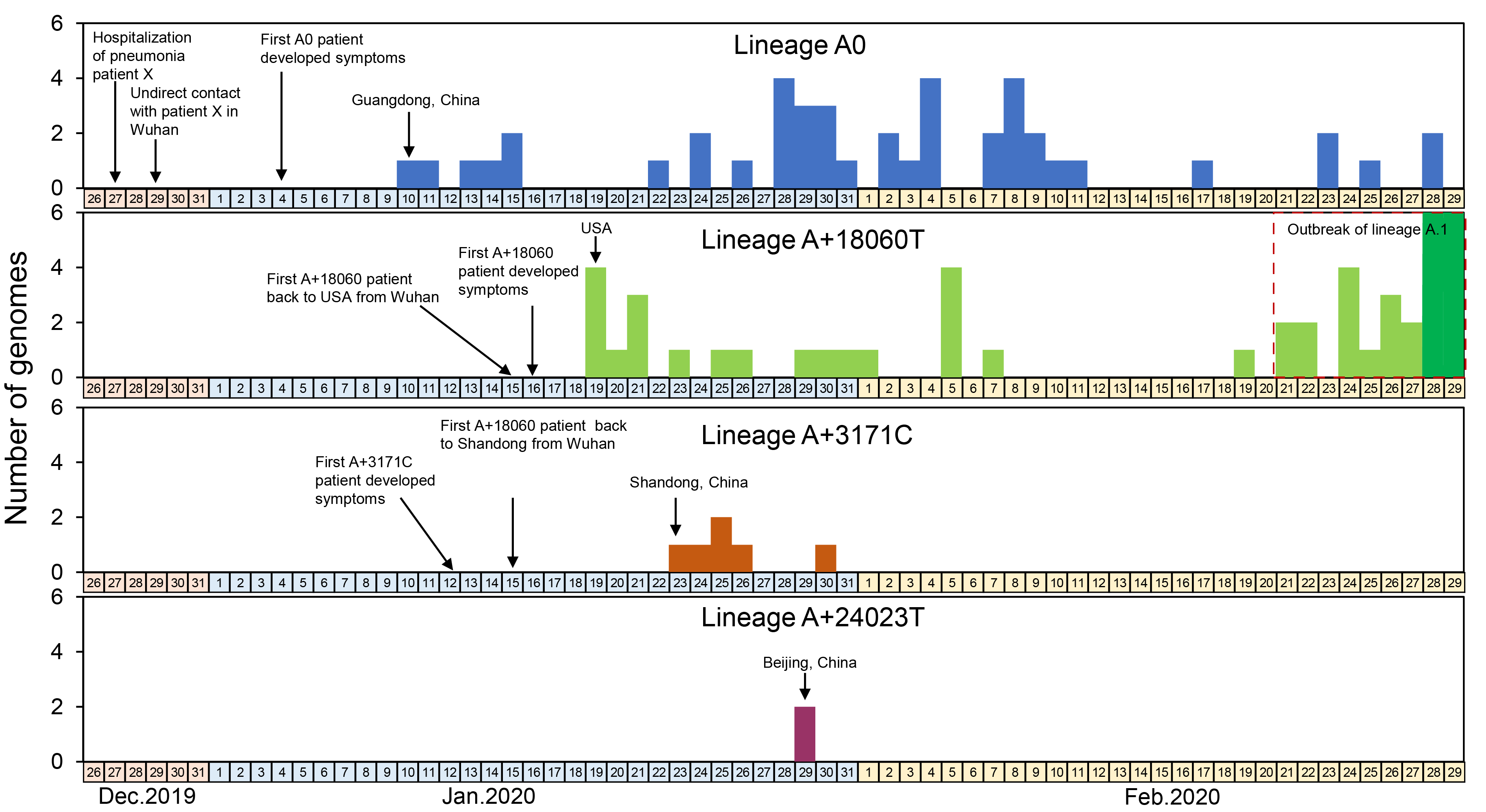

Supplement: veae020_Supp [file veae020_supp.zip › suppl_data/Supplementary Fig. 5.png]

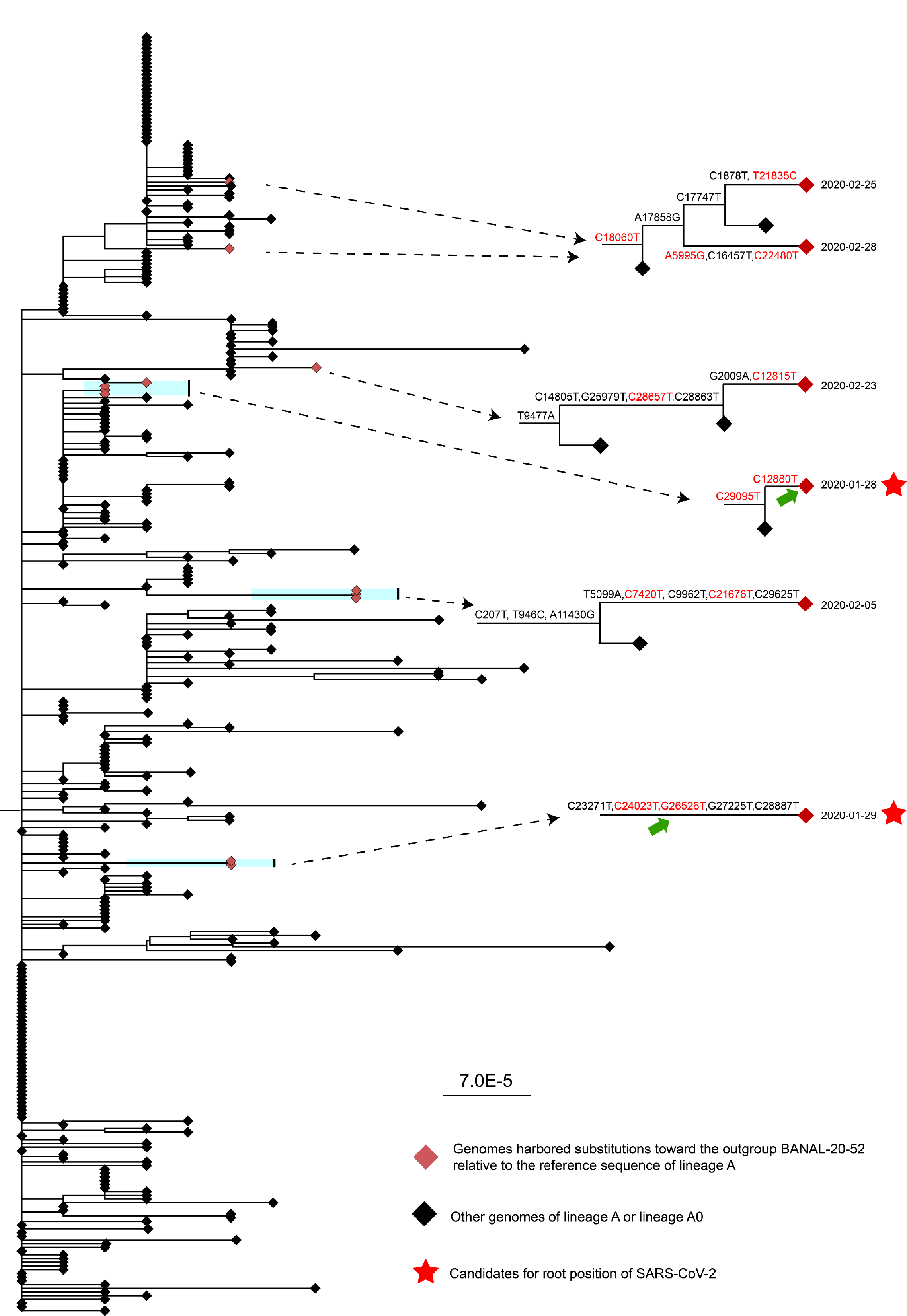

Supplement: veae020_Supp [file veae020_supp.zip › suppl_data/Supplementary Fig. 6.png]

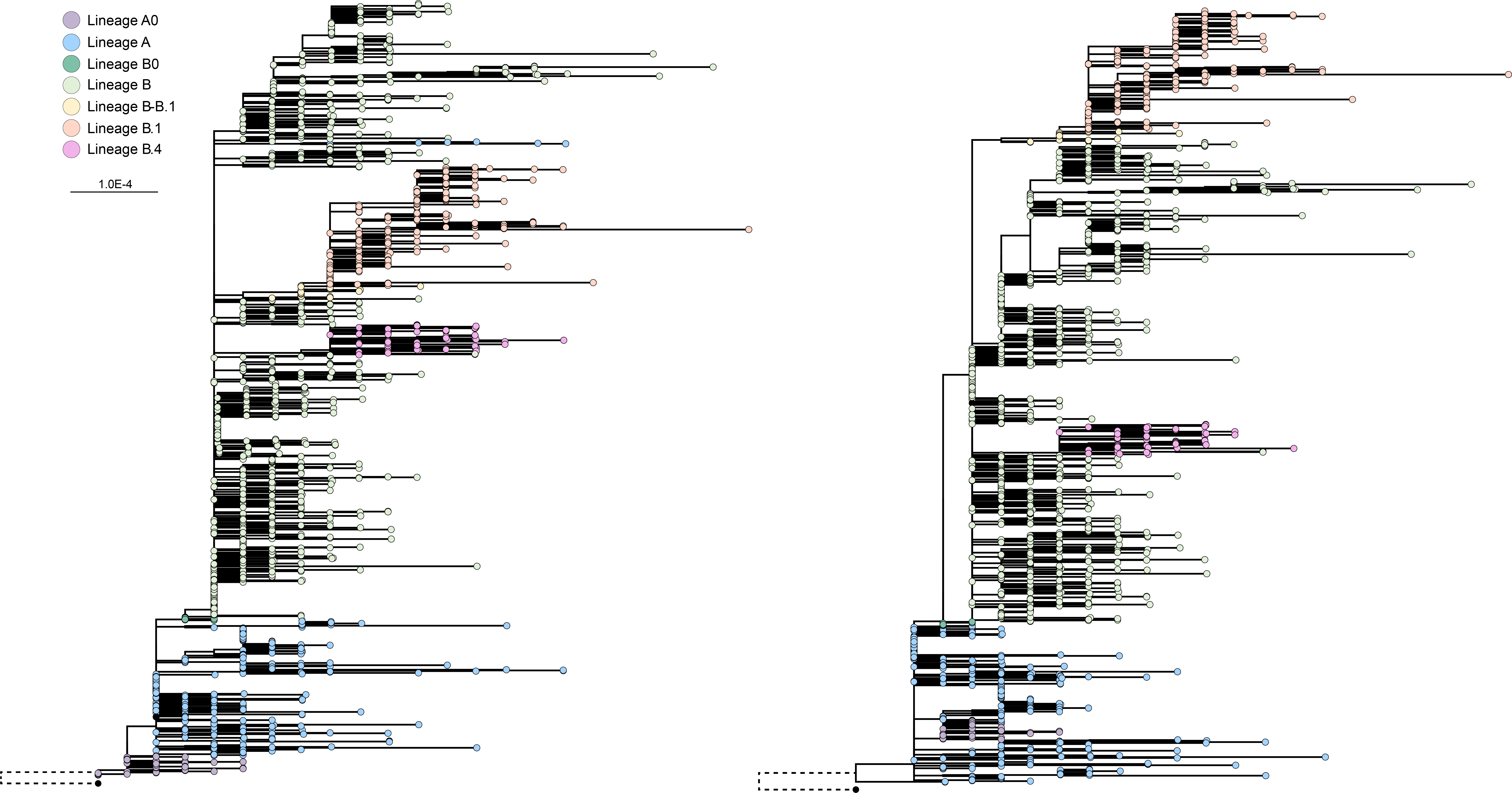

Supplement: veae020_Supp [file veae020_supp.zip › suppl_data/Supplementary Fig. 7.png]
